# MicroRNA and Hemostasis Profile of Carotid Atherosclerosis

**DOI:** 10.3390/ijms231810974

**Published:** 2022-09-19

**Authors:** Anton A. Raskurazhev, Polina I. Kuznetsova, Alla A. Shabalina, Marine M. Tanashyan

**Affiliations:** Research Center of Neurology, 125367 Moscow, Russia

**Keywords:** microRNA, cerebrovascular disease, carotid atherosclerosis, biomarkers

## Abstract

Carotid atherosclerosis (CA) is an important risk factor for ischemic stroke. We described the miRNA and hemostasis profile of patients with moderate and advanced stages of carotid atherosclerosis and elucidated potential correlations with hemostatic activation. A prospective case-control study included 61 patients with evidence of carotid atherosclerosis (via ultrasound). The study population was divided into groups depending on the degree of carotid artery stenosis: 60% or more (advanced) and <60% (moderate). All patients underwent the following blood tests: general blood test, hemostatic parameters and microRNA. Extraction of microRNA was performed using Leukocyte RNA Purification Kit (NORGEN Biotec Corp., Thorold, ON, Canada); miRNA quantification was performed via RT-PCR. Statistical analysis was performed in R programming language (v. 4.1.0) using RSudio. MicroRNA expression profile was different depending on CA degree. MiR-33a-5p/3p levels were higher in patients with ≥60% carotid stenosis (42.70 and 42.45 versus 38.50 and 38.50, respectively, *p* < 0.05). Almost complete separation can be visualized with the levels of miR-126-5p: 9.50 in the moderate CA group versus 5.25 in the advanced CA (*p* < 0.001). MiR-29-5p was higher in the moderate CA group: 28.60 [25.50;33.05] than in advanced CA group: 25.75 [24.38;29.50] (*p* = 0.086); miR-29-3p was also higher in the moderate CA group: 10.36 [8.60;14.99] than in advanced CA group: 8.46 [7.47;10.3] (*p* = 0.001). By-group pairwise correlation analyses revealed at least three clusters with significant positive correlations in the moderate CA group: miR-29-3p with factors V and XII (r = 0.53 and r = 0.37, respectively, *p* < 0.05); miR-21-5p with ADAMTS13, erythrocyte sedimentation rate and D-dimer (r = 0.42, r = 0.36 and r = 0.44, respectively, *p* < 0.05); stenosis degree with miR-33a-5p/3p and factor VIII levels (r = 0.43 (both) and r = 0.62, respectively, *p* < 0.05). Hemostasis parameters did not reveal significant changes in CA patients: the only statistically significant differences concerned factor VIII, plasminogen and (marginally significant) ADAMTS-13 and protein C. Down-regulation of miR-126-5p expression has been identified as a promising biomarker of advanced carotid atherosclerosis with high specificity and sensitivity. Correlation cluster analysis showed potential interplay between miRNAs and hemostatic activation in the setting of carotid atherosclerosis.

## 1. Introduction

Carotid atherosclerosis (CA) remains one of the leading causes of the atherothrombotic subtype of ischemic stroke, representing a major burden of disability and economic impact. Atherosclerosis represents a complex arterial disease characterized by vascular wall inflammation and remodeling, in the end resulting in the creation of atherosclerotic plaques [1]. Based on data of the Framingham cohort study, the reported prevalence of CA (defined as stenosis >50% evaluated by carotid ultrasound) in the study population was 7% in women and 9% in men [2,3]. The main factor affecting the risk of stroke among patients with CA is the degree of luminal stenosis [4]. The annual risk of stroke among patients with asymptomatic CA with <60% stenosis is ≈1%, but this risk increases to 3% to 4% for those with stenosis greater than 60% [5].

It is known that atherosclerosis is a multifactorial disease, which is based on pathology of cholesterol metabolism, various inflammatory processes and endothelial dysfunction, associated with modifiable risk factors, such as smoke, obesity, hypertension, alcohol intake, unhealthy lifestyle, stress, etc. Identification of various CA biomarkers is essential in order to develop novel diagnostic and therapeutic tools and strategies. MicroRNAs (miR) are a family of important post-transcriptional regulators of gene expression, and their involvement in the pathophysiology of cerebrovascular diseases and atherosclerosis is frequently reported. MicroRNAs may represent the above-mentioned potential diagnostic and therapeutic tools in clinical practice. MicroRNAs suppress gene expression by interacting with the 3’-untranslated region of the target messenger RNA, causing its degradation and/or blocking the translation of the gene product. An important property of miRNAs is the pleiotropy of their action, i.e., one molecule can modulate many messenger RNAs involved in various biological processes; the reverse is also true: one mRNA can be the “target” of several miRNAs [6].

**Figure 1:**
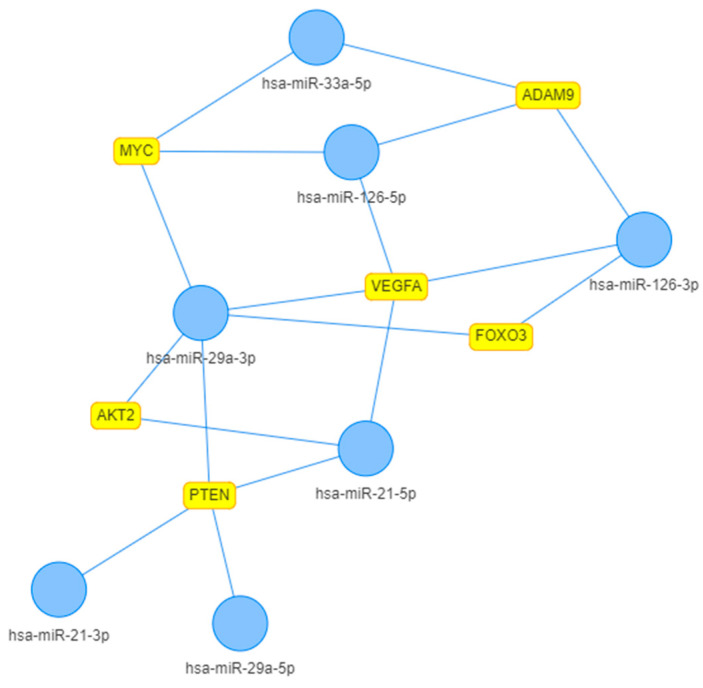
Interaction graph of selected miRNAs (blue nodes) and their target genes (yellow boxes). Built in *MIENTURNET*; network layout type ‘GEM’.

Previously, we studied a number of miRNAs (namely miR-126-5p, miR-126-3p, miR-29a-5p, miR-29a-3p, miR-33a-5p, miR-33a-3p, miR-21-5p and miR-21-3p) involved in CA pathogenesis [7]. When compared with otherwise healthy controls, patients with CA demonstrated a different pattern of miRNA expression. In order to visualize interaction networks between these selected miRNAs and target genes, we built an interaction graph (via *MIENTURNET*) with only strongly validated targets, a minimum three of which were shared, and a 0.05 threshold for the adjusted *p* value (Figure 1) [8].

All the target genes shown in Figure 1 are implicated in various mechanisms of atherosclerosis development and progression: pro-inflammatory cell interactions (ADAM9), dysregulation of vascular homeostasis (FOXO3), cell proliferation and angiogenesis (VEGFA/MYC pathway), vascular injury and remodeling (PTEN) and macrophage activation (AKT2) [9,10,11,12,13].

It has also been shown that hemostasis pathology is associated with the development of carotid atherosclerosis [14,15]. Thus, it could be interesting not only to evaluate the relationship of CA with miRNA expression levels but also to observe and analyze possible interactions between miRNAs and hemostasis biomarkers depending on the severity of CA.

## 2. Results

All patients’ clinical and demographic data are presented in Table 1. Both groups were comparable by gender and age. More patients in the adCA group had prior stroke (twelve versus eight), but the difference was not statistically significant. The most common comorbidity in both groups was arterial hypertension. The major cardiovascular risk factor was similarly distributed between groups, but the prevalence of other sites of atherosclerosis (predominantly of the lower limbs’ arteries) was significantly higher in patients with advanced CA (*p* < 0.001). Both internal carotid arteries were involved in 49.2% of patients. Coronary heart disease burden was also higher in this group. 

Routine laboratory work-up (Table 2) did not demonstrate any statistically significant difference between the two groups. Total cholesterol and LDL levels were relatively low, perhaps due to high rates of statin treatment. A moderate increase in erythrocyte sedimentation rate was also noted. 

**Figure 2:**
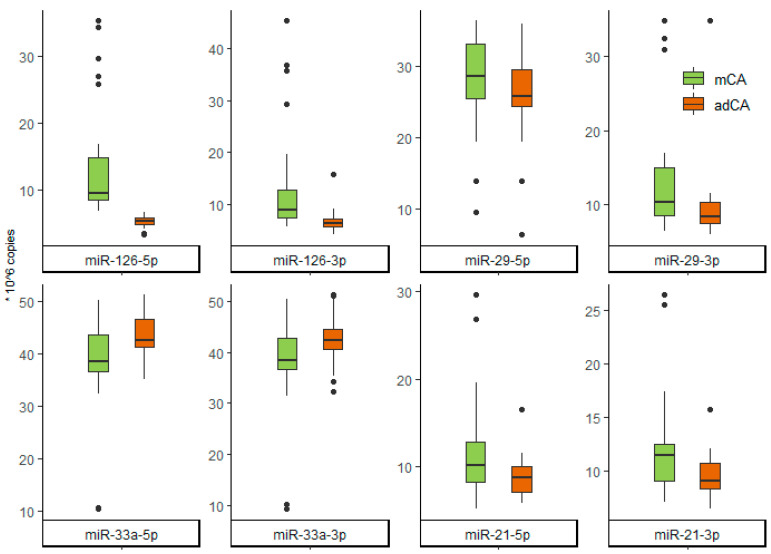
Box plot of microRNA expression levels in the study population (×10^6^ copies). The thick horizontal line represents the median; thin horizontal lines the 1st and 3rd quartile; vertical lines indicate the spread (outliers (dots) are not removed).

MicroRNA expression profile was profoundly different depending on CA degree (Table 3 and Figure 2). MiR-33a-5p/3p levels were the only ones higher in patients with ≥60% carotid stenosis (42.70 and 42.45 versus 38.50 and 38.50 respectively, *p* < 0.05). Almost complete separation can be visualized with the levels of miR-126-5p: 9.50 in the mCA group versus 5.25 in the adCA (*p* < 0.001). All other microRNAs were also lower in advanced CA patients.

Hemostasis parameters did not reveal significant changes in CA patients: the only statistically significant differences concerned factor VIII, plasminogen and (marginally significant) ADAMTS-13 and protein C (Table 4). 

**Figure 3:**
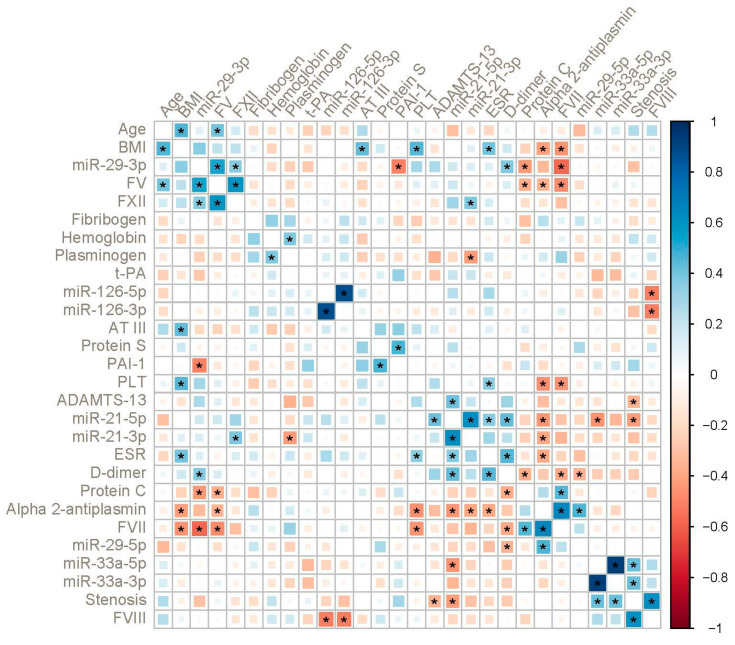
Correlogram in patients with mild-to-moderate CA with hierarchical clustering. The color indicates the direction and strength of correlation (Spearman’s ρ). Asterisks indicate statistically significant correlations (*p* < 0.05).

**Figure 4:**
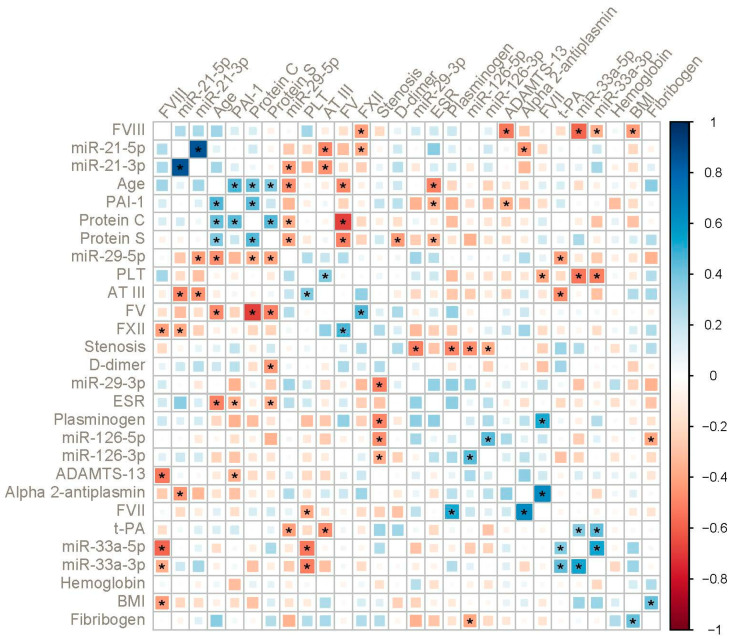
Correlogram in patients with advanced CA with hierarchical clustering. The color indicates the direction and strength of correlation (Spearman’s ρ). Asterisks indicate statistically significant correlations (*p* < 0.05).

In order to elucidate possible biomarker interactions, we performed by-group pairwise correlation analyses, which were visualized by clustered correlograms (Figure 3 and Figure 4; as for clustering method, see ‘Statistics’ in the ‘Materials and Methods’ section). In the mCA group, we can identify at least three clusters with significant positive correlations: miR-29-3p with factors V and XII (r = 0.53 and r = 0.37, respectively, *p* < 0.05); miR-21-5p with ADAMTS13, ESR and D-dimer (r = 0.42, r = 0.36 and r = 0.44, respectively, *p* < 0.05); stenosis degree with miR-33a-5p/3p and factor VIII levels (r = 0.43 (both) and r = 0.62, respectively, *p* < 0.05). Prominent negative correlations included: factor V levels with protein C, alpha-2-antiplasmin and factor VII (r = −0.37, r= −0.36 and r = −0.48, respectively, *p* < 0.05); factor VIII with miR-126-5p/3p (r = −0.52, *p* < 0.05); alpha-2-antiplasmin with miR-21-5p/3p (r = −0.44 and r = −0.40, respectively, *p* < 0.05).

In the advanced CA group, different clusters were identified. Age was positively associated with PAI-1, protein C and S levels (r = 0.46, r = 0.43 and r = 0.38, respectively, *p* < 0.05); factors V and XII were also positively correlated (r = 0.47, *p* < 0.05). Of the miRs, the only noticeable positive correlation was that of miR-33a-5p/3p with t-PA levels (r = 0.37, r = 0.43, respectively, *p* < 0.05). Significant negative correlations were observed for miR-29-5p with miR-21-3p, age, protein C and S levels and t-PA (r = −0.43, r = −0.46, r = −0.37, r = −0.41 and r = −0.42, respectively, *p* < 0.05). Factor V levels were inversely correlated with protein C and S levels (r = −0.69 and r = −0.51, respectively, *p* < 0.05). 

**Figure 5:**
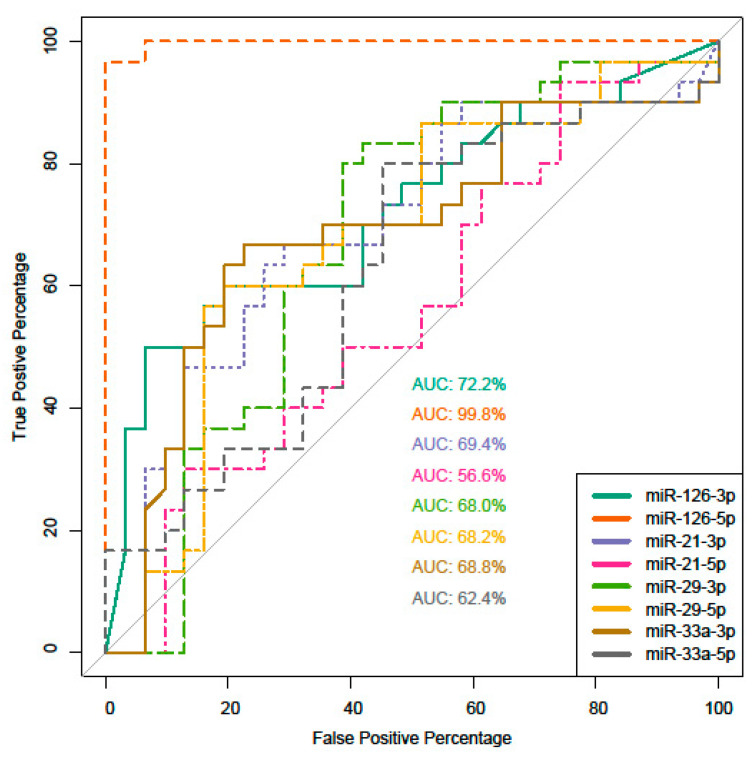
Receiver operating characteristic (ROC) curves for different microRNAs.

A random forest analysis performed for each miRNA in terms of classifying the advanced CA group demonstrated high levels of specificity and sensitivity for expression levels of miR-126-5p (Figure 5). No multivariate analysis was performed due to near complete separation with miR-126-5p as a covariate (see below in the ‘Discussion’ section).

## 3. Discussion

Carotid atherosclerosis (CA) is one of the major risk factors for ischemic stroke, and it is a diagnosis readily available in routine clinical practice via ultrasound. Yet, the complex interplay between various biomarkers (endothelial function, hemostasis, lipid profile, etc.) leading to atherosclerosis progression and eventually (in certain patients) to overt cerebrovascular disease is still unclear. In our study, we hypothesized that patients with different stages of CA would have distinct profiles of microRNA expression and hemostatic activity. 

MicroRNAs are an important part of epigenetic regulation and have been proven to be implicated in a wide range of cardiovascular disorders [16]. Almost all miRNAs in our study were differentially expressed depending on the degree of CA, with miR-126-5p nearly two-fold up-regulated in patients with moderate CA. 

The miR-126 duplex is one of the most highly expressed in endothelial cells and is instrumental for angiogenesis and vascular integrity, featuring strand-specific functions and homeostasis [17]. It is understood that miR-126-5p is up-regulated by laminar flow in arterial endothelial cells to limit atherosclerosis in areas of high shear stress [18]. This may be the reason why patients with advanced CA in our study demonstrated severe down-regulation of both strands of miR-126, leaving open the question of whether these alterations were a consequence of atherosclerotic plaque formation or played a mediating role. These results are corroborated by Santovito et al., who found reduced levels of miR-126-5p in human plaques in areas of disturbed flow [17].

Another family of miRNAs that was significantly down-regulated in advanced CA was miR-21-5p/3p. These miRNAs target PPARα, a key regulator of lipid-metabolizing enzymes, and may promote angiogenesis and suppress apoptosis in endothelial cells post hypoxia [19,20]. Experimental studies have also shown that overexpression of miR-21 increased NO production in human endothelial cells, thus contributing to attenuation of atherosclerosis-induced endothelial dysfunction [21]. The expression of miR-21, along with another from our study—miR-29—was, nevertheless, shown to be up-regulated in circulating CD4 + T cells of patients with atherosclerosis obliterans, a finding that contradicts our results [22].

The only up-regulated miRNAs in advanced CA in our study were both strands of miR-33a, which synergistically promote cellular cholesterol accumulation in macrophages. Blocking miR-33-5p has resulted in rising HDL levels, which may be attributed to targeting ABCA1 in the liver [23].

The hemostasis profile was less distinct between the two study groups; one of the more prominent findings was increased factor VIII activity in advanced CA. Along with von Willebrand factor (which acts as its carrier protein), its role has been discussed in endothelial dysfunction and atherosclerosis [24]. Our results show that factor VIII activity was negatively correlated with miR-126 levels only in the moderate CA group. This may suggest a potentially atheroprotective setting in the early stages of CA, which decreases as atherosclerosis progresses. In the latter group, factor VIII was inversely associated with miR-33a-5p/3p levels, an interesting finding considering their putative proatherogenic nature.

Another hemostasis marker—ADAMTS13 (*a disintegrin and metalloproteinase with a thrombospondin type 1 motif, member 13*)—was marginally significantly lower in the adCA group. It has been previously shown that low ADAMTS13 plasma levels are associated with an increased risk of arterial thrombosis, including cerebrovascular disease [25]. In our study, in the mCA group, ADAMTS13 was part of a cluster positively correlated with miR-21, a potentially atheroprotective agent, while, in the adCA patients, it was negatively correlated with levels of PAI-1 and FVIII.

## 4. Materials and Methods

### 4.1. Study Population and Clinical Data

This was a prospective case-control study that included 61 patients (median age 66.0 years, 55.7% male) with evidence of carotid atherosclerosis (CA, via ultrasound (adopting the NASCET criteria)). The recruitment took place from January 2020 until March 2021 at Research Center of Neurology, Moscow, Russia. The study population was then divided in two groups depending on the degree of carotid artery stenosis: 60% or more (advanced CA, adCA) and <60% (moderate CA, mCA). History of previous stroke, smoking, arterial hypertension, coronary heart disease, previous myocardial infarction, diabetes mellitus, atrial fibrillation, evidence of atherosclerosis in other locations and concurrent treatment (acetylsalicylic acid, anticoagulants and statins) was noted. Patients with malignancies, current infectious diseases, decompensated somatic pathology (including severe renal/hepatic disease), autoimmune disorders and stroke/myocardial infarction within 6 months were not included in this study.

### 4.2. Laboratory Analyses

All patients underwent the following blood tests: general blood test: HB (g/l), RBC (10^12^/L), PLT (10^9^/L), WBC (10^9^/L) was carried out on an automatic hematological analyzer Nihon Kohden MEK-7222 (Nihon Kohden Corp., Tokyo, Japan), ESR (mm/h) was measured by the Panchenkov method; plasma hemostasis indicators: fibrinogen concentration (FG, g/l), D-dimer level (ng/mL), plasma coagulation factors V, VII, VIII, XII (%) activity, activity anticoagulant system: antithrombin-III (AT-III, %), proteins C (PC, %), S (PS, %) and fibrinolysis: plasminogen (PG, %), a2-antiplasmin (PL-IN, %) were determined by generally accepted methods on automatic coagulometer ACL Elite Pro (Werfen, Bedford, MA, USA) using reagents Instrumentation Laboratory (Werfen, Bedford, MA, USA) and RENAM (SPD RENAM, Moscow, Russia). Study of tissue plasminogen activator (t-PA, ng/mL), tissue plasminogen activator inhibitor (PAI-1, ng/mL), ADAMTS-13 metalloproteinase (ADAMTS-13, mcg/mL) in blood plasma was carried out by the solid-phase enzyme immunoassay (ELISA) on the Victor 2 (PerkinElmer, Waltham, MA, USA) and ‘Real-best’ spot readers (VectorBest, Novosibirsk, Russia) using Technoclone reagent kits (Technoclone, Vienna, Austria). Lipid profile (cholesterol (mmol/L), triglycerides TG (mmol/L), HDL (mmol/L), LDL (mmol/L), were measured on an automatic biochemical analyzer Konelab 30i (Thermo Scientific, Waltham, USA) using Randox reagent kits (Randox Laboratories, Crumlin, UK).

### 4.3. MicroRNA Extraction

We described microRNA extraction and quantification in detail previously [26]. Briefly, extraction of microRNA was performed using Leukocyte RNA Purification Kit (NORGEN Biotec Corp., Thorold, ON, Canada) according to modified manufacturer protocol. The PCR was performed starting with the reverse transcription step.

The following reagents and equipment have been used:

- Validated 20X primers for has-miR: miR-126-5p, miR-126-3p, miR-29-5p, miR-29-3p, miR-33a-5p, miR-33a-3p, miR-21-5p, miR-21-3p (ThermoFischerScientific, Waltham, MA, USA)
- Leukocyte RNA Purification Plus Kit (NORGEN Biotec Corp., Thorold, ON, Canada)
- TaqMan™ Advanced miRNA cDNA Synthesis Kit (Applied Biosystems™, Thermo Fisher Scientific, Waltham, MA, USA)
- Real-time CFX96 Touch amplifier (BioRaD, Hercules, CA, USA)

### 4.4. Statistics

Statistical analysis was performed in R programming language (v. 4.1.0) using RSudio (version 1.4.1717) and the following downloadable packages: ‘tidyverse’, ‘reshape’, ‘corrplot’, ‘pROC’, ‘randomForest’, ‘Hmisc’. Nonparametric tests were implemented. Discrete data are presented as frequency (%), continuous—as median (first quartile; third quartile). Comparison of two proportions was conducted with the Pearson z test with continuity correction. The Wilcoxon–Mann–Whitney test was used for two-sample comparisons. Relationship between variables of interest was analyzed using Spearman’s rank correlation coefficient (ρ). By-group correlation matrices were plotted with the variables ordered by hierarchical clustering (Ward’s minimum variance method). A random forest algorithm (number of trees = 500) was applied to the expression levels of all 8 microRNAs for classification analysis based on CA subdivision (≥60% or <60%). The resulting predicted probabilities were used to build receiver operating characteristic (ROC) curves and calculate the area under the curve (AUC) for each microRNA. All statistical tests were two sided and results were deemed statistically significant if *p* value was < 0.05.

## 5. Conclusions

Carotid atherosclerosis (CA) is one of the leading causes of ischemic stroke, the risk of which increases with the advancement of CA. In our study, we described the miRNA and hemostasis profile of patients with moderate and advanced stages of CA. Down-regulation of miR-126-5p expression has been identified as a promising biomarker of advanced CA with high specificity and sensitivity. Correlation cluster analysis showed potential interplay between miRNAs and hemostatic activation in the setting of carotid atherosclerosis, yet further validation and prospective studies are required. To our knowledge, this is the first study to explore miRNAs and hemostasis biomarkers in patients with carotid atherosclerosis.

## 6. Limitations

Our study has several limitations, among which the most prominent is the small cohort size (yet, in many reviewed publications on miRNAs, sample sizes are relatively small); another is the potential for selection bias (the patients were recruited at one center). We defined the groups according to the maximal degree of stenosis in the carotid arteries, but, on account of certain definition difficulties, did not take into account the number of involved carotid/vertebral arteries (possibly in the mild-to-moderate CA group), which may be an important factor influencing miRNA expression. The absence of a pure control group may also be a limiting factor in this study, yet our main goal was to identify miRNA expression and hemostasis biomarkers depending on degree of CA. A near complete separation was found for the expression levels of miR-126-5p, which precluded us from performing multivariable logistic regression and also yielded high specificity and sensitivity values in ROC curve analysis. This may potentially suggest flaws in miRNA quantification, but, in our opinion, justifies further replication and validation studies. Both miRNA expression and hemostasis biomarkers could have been influenced by concurrent medications (including ASA and statin treatment). Nevertheless, we consider the groups to be evenly comparable regarding most clinical and demographic characteristics.

## Figures and Tables

**Table 1:** Clinical and demographic characteristics of the study population.

	mCA (n = 31)	adCA (n = 30)	Study Population (n = 61)	*p* *
Male, n (%)	17 (54.8)	17 (56.7)	34 (55.7)	0.99
Age, years (median [Q1;Q3])	65.0 [61.5;70.0]	68.5 [61.3;71.8]	66.0 [61.0;71.0]	0.19
BMI, kg/m^2^ (median [Q1;Q3])	27.1 [25.0;29.5]	27.2 [26.3;29.2]	27.2 [25.5;29.4]	0.60
Smokers, n (%)	8 (25.8)	13 (43.3)	21 (34.4)	0.24
Stroke, n (%)	8 (25.8)	12 (40.0)	20 (32.8)	0.36
AH, n (%)	27 (87.1)	29 (96.7)	56 (91.8)	0.37
CHD, n (%)	6 (19.4)	18 (60.0)	24 (39.3)	0.003
MI, n (%)	3 (9.7)	7 (23.3)	10 (16.4)	0.27
DM, n (%)	11 (35.5)	15 (50.0)	26 (42.6)	0.38
Carotid surgery, n (%)	2 (6.5)	24 (80.0)	26 (42.6)	<0.001
Atherosclerosis of other locations, n (%)	10 (32.3)	26 (86.7)	36 (59.0)	<0.001
AF, n (%)	3 (9.7)	4 (13.3)	7 (11.5)	0.96
ASA, n (%)	26 (83.9)	29 (96.7)	55 (90.2)	0.21
Anticoagulants, n (%)	5 (16.1)	8 (26.7)	13 (21.3)	0.49
Statins, n (%)	22 (71.0)	28 (93.3)	50 (82.0)	0.05

*—difference between mCA and aCA. mCA—moderate carotid atherosclerosis; adCA—advanced carotid atherosclerosis; BMI—body mass index; AH—arterial hypertension; CHD—coronary heart disease; MI—myocardial infarction; DM—type 2 diabetes mellitus; AF—atrial fibrillation; ASA—acetylsalicylic acid therapy.

**Table 2:** Routine laboratory findings in the study population.

	mCA (n = 31)	adCA (n = 30)	Study Population (n = 61)	*p* *
TC, mmol/L	4.9 [4.4;6.5]	4.6 [4.2;5.4]	4.8 [4.2;6.0]	0.25
LDL, mmol/L	2.0 [1.0;2.7]	2.0 [1.5;2.7]	2.0 [1.4;2.7]	0.36
HDL, mmol/L	1.7 [1.3;2.1]	1.7 [1.4;2.1]	1.7 [1.3;2.1]	0.78
TG, mmol/L	1.8 [1.1;2.0]	1.3 [0.9;2.0]	1.4 [1.0;2.0]	0.30
Hb, g/L	122 [113;130]	116 [110;128]	119 [110;130]	0.28
RBC, * 10^12^/L	4.1 [4.0;4.4]	4.4 [4.0;4.8]	4.2 [4.0;4.8]	0.13
WBC, * 10^9^/L	6.7 [6.2;7.5]	7.2 [6.4;8.3]	6.8 [6.4;7.5]	0.15
Platelets, * 10^9^/L	215 [172;280]	215 [150;253]	215 [165;260]	0.45
ESR, mm/h	20 [16;23]	21 [14;26]	20 [16;26]	0.59

*—difference between mCA and adCA. TC—total cholesterol; LDL—low-density lipoproteins; HDL—high-density lipoproteins; TG—triglycerides; Hb—hemoglobin; RBC—red blood cells; WBC—white blood cells; ESR—erythrocyte sedimentation rate.

**Table 3:** MicroRNA expression in the study population.

	mCA (n = 31)	adCA (n = 30)	*p*
miR-126-5p	9.50 [8.42;14.80]	5.25 [4.76;5.68]	<0.001
miR-126-3p	8.95 [7.35;12.70]	6.36 [5.59;7.24]	<0.001
miR-21-5p	10.20 [8.26;12.90]	8.73 [7.15;10.05]	0.048
miR-21-3p	11.45 [9.15;12.57]	9.14 [8.37;10.73]	0.003
miR-29-5p	28.60 [25.50;33.05]	25.75 [24.38;29.50]	0.086
miR-29-3p	10.36 [8.60;14.99]	8.46 [7.47;10.3]	0.001
miR-33a-5p	38.50 [36.55;43.70]	42.70 [41.30;46.60]	0.015
miR-33a-3p	38.50 [36.60;42.70]	42.45 [40.55;44.60]	0.018

**Table 4:** Hemostasis markers in the study population.

	mCA (n = 31)	adCA (n = 30)	*p*
D-dimer, ng/mL	360 [185;510]	350 [260;480]	0.42
Fibrinogen, g/L	3.71 [3.27;4.19]	3.63 [3.16;4.07]	0.53
AT III, %	91.5 [84.0;101.0]	87.7 [84.0;96.0]	0.29
Protein C, %	72.3 [63.3;78.0]	66.5 [62.0;70.4]	0.06
Protein S, %	70.0 [60.5;82.2]	71.0 [60.5;72.4]	0.85
Plasminogen, %	69.0 [64.2;88.3]	82.0 [68.5;98.0]	0.04
Alpha 2-antiplasmin, %	95.0 [92.4;104.0]	100.0 [94.3;107.0]	0.17
FV, %	76 [68;89]	76 [68;97]	0.76
FVII, %	67 [64;98]	85 [71;112]	0.10
FVIII, %	110 [89;142]	160 [141;185]	<0.001
FXII, %	84 [68;106]	86 [68;101]	0.78
ADAMTS13, ug/ml	0.88 [0.76;1.08]	0.82 [0.67;0.99]	0.08
t-PA, ng/mL	2.9 [2.5;3.6]	2.77 [2.28;3.58]	0.69
PAI-1, ng/mL	47.4 [31.35;49.9]	44.5 [19.4;55.2]	0.55

AT—antithrombin III; FV—factor V (Leiden); FVII—factor VII; FVIII—factor VIII; FXII—factor XII; ADAMTS-13—a disintegrin and metalloproteinase with a thrombospondin type 1 motif, member 13; t-PA—tissue plasminogen activator; PAI-1—plasminogen activator inhibitor-1.

## Data Availability

The data that support the findings of this study are available from the corresponding author, P.K., upon reasonable request.
